# High-throughput three-dimensional visualization of root system architecture of rice using X-ray computed tomography

**DOI:** 10.1186/s13007-020-00612-6

**Published:** 2020-05-11

**Authors:** Shota Teramoto, Satoko Takayasu, Yuka Kitomi, Yumiko Arai-Sanoh, Takanari Tanabata, Yusaku Uga

**Affiliations:** 1 Institute of Crop Science, National Agriculture and Food Research Organization, Tsukuba, Ibaraki 305-8518 Japan; 2 Kazusa DNA Research Institute, Kisarazu, Chiba 292-0818 Japan

**Keywords:** Image processing, *Oryza sativa*, Plant root, Root plasticity, RSAvis3D, X-ray CT

## Abstract

**Background:**

X-ray computed tomography (CT) allows us to visualize root system architecture (RSA) beneath the soil, non-destructively and in a three-dimensional (3-D) form. However, CT scanning, reconstruction processes, and root isolation from X-ray CT volumes, take considerable time. For genetic analyses, such as quantitative trait locus mapping, which require a large population size, a high-throughput RSA visualization method is required.

**Results:**

We have developed a high-throughput process flow for the 3-D visualization of rice (*Oryza sativa*) RSA (consisting of radicle and crown roots), using X-ray CT. The process flow includes use of a uniform particle size, calcined clay to reduce the possibility of visualizing non-root segments, use of a higher tube voltage and current in the X-ray CT scanning to increase root-to-soil contrast, and use of a 3-D median filter and edge detection algorithm to isolate root segments. Using high-performance computing technology, this analysis flow requires only 10 min (33 s, if a rough image is acceptable) for CT scanning and reconstruction, and 2 min for image processing, to visualize rice RSA. This reduced time allowed us to conduct the genetic analysis associated with 3-D RSA phenotyping. In 2-week-old seedlings, 85% and 100% of radicle and crown roots were detected, when 16 cm and 20 cm diameter pots were used, respectively. The X-ray dose per scan was estimated at < 0.09 Gy, which did not impede rice growth. Using the developed process flow, we were able to follow daily RSA development, i.e., 4-D RSA development, of an upland rice variety, over 3 weeks.

**Conclusions:**

We developed a high-throughput process flow for 3-D rice RSA visualization by X-ray CT. The X-ray dose assay on plant growth has shown that this methodology could be applicable for 4-D RSA phenotyping. We named the RSA visualization method ‘RSAvis3D’ and are confident that it represents a potentially efficient application for 3-D RSA phenotyping of various plant species.

## Background

Roots are essential plant organs necessary for taking up water and nutrients from the soil. To absorb water and nutrients effectively, plants develop roots with different forms and functions, such as primary and lateral roots, and root hairs. The spatial distribution of such different root types in the ground is defined as the root system architecture (RSA) [1]. Plants adjust their RSA in response to the changing environment; for example, in the case of nutrient absorption, when roots encounter soil zones or patches containing rich nutrients, such as nitrate, ammonium, phosphate, or potassium, plants increase the lateral root biomass in the area, in order to increase their nutrient uptake capacity [2]. In contrast, plants inhibit root growth locally to avoid entering undesirable regions containing poor phosphorus levels, [3] or heavy metals at toxic levels [4]. Deficiency of nutrients other than phosphorus causes various RSA changes as well [5, 6]. In the case of abiotic stress responses, a meta-analysis of the effect of drought stress on root phenotypes showed that drought stress led to decreased root length and root length density, and increased the diameter, root–shoot mass ratio, and root cortical aerenchyma [7]. Such RSA flexibility in response to environmental stimuli has been used to optimize plant growth and development [2–7]. Understanding the responses of plants to stimuli is beneficial in developing crop cultivars that adapt to environmental stresses.

Isolating the genes or quantitative trait loci (QTLs) affecting RSA plasticity is a useful technique to apply to crop breeding [8]. Genetic analysis approaches are preferably performed on large populations, as the larger population size increases the power and accuracy of QTL detection [9, 10]. Population size is determined by line number and repetition. Many QTL mapping studies have used progeny population consisting of over 100 lines, derived from a cross between bi-parental lines [10] and several repetitions, to detect QTLs associated with low-heritability traits. For example, QTLs associated with root morphological traits were isolated by using 600 individual rice plants (*Oryza sativa*), consisting of 120 lines with five repetitions [11]. Therefore, genetic analysis such as QTL mapping requires a high-throughput RSA phenotyping system to deal with large populations.

Under field conditions, many RSA phenotyping methods have been developed and applied [12]. Some of them have a high throughput and are used for genetic analysis. For example, digging up the roots in the soil [13] is a simple and high-throughput method, being applied for genome-wide association studies to isolate loci affecting root architectural traits in *Brassica napus* [14]. Other popular field methods, such as monolith, auger, profile wall, and glass wall methods [12], involve longer processing times.

It is generally accepted that such physical field methods are destructive and can be used to observe only a portion of the soil-based RSA [15]. Being destructive, such methods are difficult to apply to gene isolation studies, or to QTLs affecting RSA plasticity. Under laboratory conditions, there are many reports indicating that high-throughput RSA phenotyping, using a combination of agar media and Arabidopsis (*Arabidopsis thaliana*), has been widely used for genes or QTL isolation. For example, a split-root experimental system, using segmented vertical agar plates, revealed that the nitrate transporter NRT1.1 [16], and the ammonium transporter AMT1;3 [17], play important roles in nitrate- and ammonium-induced root colonization, respectively. Two robust QTLs affecting the RSA response to osmotic stress have been detected in a growth assay using agar media, using a recombinant inbred population combining two Arabidopsis ecotypes, namely, Landsberg *erecta* and Columbia [18]. Although most media such as agar are transparent, which enable non-destructive RSA phenotyping, opaque soil media would need to be used if field conditions are to be mimicked.

Recently, X-ray computed tomography (CT) and magnetic resonance imaging (MRI) have been employed to observe root growth dynamics in soil [19]. X-ray CT has the potential for a wide range of applications in plant phenotyping, as non-medical X-ray CT scanners are available at lower equipment cost compared with MRI equipment [19]. X-ray CT visualizes roots in the soil using the attenuation differences caused by X-rays between materials. For X-ray CT imaging, signal data from multi-angle projections are used to compute densitometric slice images (reconstructions), which are stacked to construct 3-D densitometric volumes. Using these processes, soil RSA can be observed nondestructively [20].

X-ray CT has been used in various studies for nondestructive root observation in the soil. For example, X-ray CT imaging revealed the interaction of two individual plant roots in *Populus tremuloides* and *Picea mariana* [21], development of porous architecture at the root–soil interface in a tomato (*Solanum lycopersicum*) [22], and interactions between roots and phosphate fertilizer in wheat (*Triticum aestivum*) [23]. However, high-throughput root phenotyping, using X-ray CT for investigating the time-course of 3-D or 4-D RSA development, has not been intensively studied to date, despite its importance in revealing RSA flexibility in the soil. This has been partly due to the long scanning and reconstruction times, small scanning areas, and laborious processes involved in the root segmentation in X-ray CT imaging [24]. As a 4-D study requires repeated scanning of identical samples, shorter scanning and processing times are required.

In recent decades, scanning and reconstruction times have been reduced, by optimizing both hardware and software [25], however the root segmentation process remains laborious. Root segments in 3-D densitometric data are mainly isolated using three methods: manual, semi-automatic, and fully automatic. In the manual method, root segments in the 3-D data are manually selected by using simple drawing tools, such as polylines [23]. The manual method is simple and applicable for analyzing soil with complex texture but selecting the region of interest requires a long time. The semi-automatic method employs a simple algorithm for root segmentation. Thresholding and region growing isolate the root segments, based on the image intensity differences between roots and the soil [22, 24]. Particle tracking is a more complex algorithm, but is widely used for root segmentation [26–28]. Because most plant roots penetrate the soil vertically, or along the z-axis, the roots in horizontally sliced images are regarded as particles, in this method, which are tracked along the z-axis. The fully automatic method automatically isolates root segments, with no manual operation or operators needed, making it potentially applicable in high-throughput, 4-D studies.

To date, there have been a small number of applications of the fully automatic method to plant root segmentation, one being a method based on deep neural networks for root segmentation [29], and another on feature detection focusing on the tubular shapes of roots [30]. The former is a developing technique for root segmentation in soil and has potential for application to 4-D root phenotyping [29]. The latter was designed based on a medical image analysis technique for detecting blood vessels [30], which is widely used in the medical field [31]. Because the target of the latter method is roots, including laterals, growth pot diameter has to be relatively small (70 mm), and the X-ray dose for scanning one plant sample has to be relatively high (2.5 Gy). To conduct high-throughput RSA phenotyping using X-ray CT, a fully automatic method applicable to large pot diameters, using rapid scanning, and low X-ray doses, is needed.

Here, we have developed a process flow for high-throughput, 3-D RSA visualization that is suitable for 4-D RSA phenotyping. In this study, we used rice as a representative monocotyledonous crop with a fibrous root system [32], and focused on radicle and crown roots, which form the main RSA skeleton. To achieve rapid CT scanning over a large scanning area, cultivation and CT scanning conditions were determined, and then, to improve root isolation efficiency, a simple algorithm of fully automated root isolation was constructed. To make the flow acceptable for repeated CT scanning, the effect of X-ray doses on plant growth was evaluated, and 4-D RSA development of an upland rice over 3 weeks was visualized. To our knowledge, this is the first time that a 4-D RSA visualization system for crops, verified with respect to not only cultivation and scanning conditions, but also in relation to the effect of X-ray doses on plants, has been reported.

## Results

### Conditions for X-ray CT scanning

A scanning time of 10 min for each plant sample was determined as the X-ray CT condition for application to high-throughput crop RSA phenotyping. Because additional time was required for machine operation, X-ray generator start-up, and saving CT images, 15 min was the actual elapsed time needed per single sample. Thus, 32 samples could be processed in an 8-h day, and 160 individuals, which is sufficient to perform genetic analysis, could be scanned in a week. To observe RSA development in rice continuously, until the roots reached the pot wall, pot diameter and depth were set as 20 cm and 25 cm, respectively, based on the maximum size that could be scanned by the detector of the CT scanner used in this study.

To obtain CT volumes showing clear root shapes, we determined the best soil substrate for CT scanning. We used an upland rice cultivar Kinandang Patong (KP) as the test sample, as upland rice usually has thicker roots than lowland rice [33], making it relatively easy to isolate the root segments from the CT volumes. To select a soil substrate suitable for rice root CT scanning, we examined the CT images of KP grown in five different soil substrates: calcined clay, volcanic ash soil, andosol, alluvial soil, and sand (Additional file 1). Among these five types, calcined clay gave the clearest root imagery in the CT process (Additional file 2). Based on this result, we decided to use calcined clay in our rice root CT scanning experiments.

**Fig. 1:**
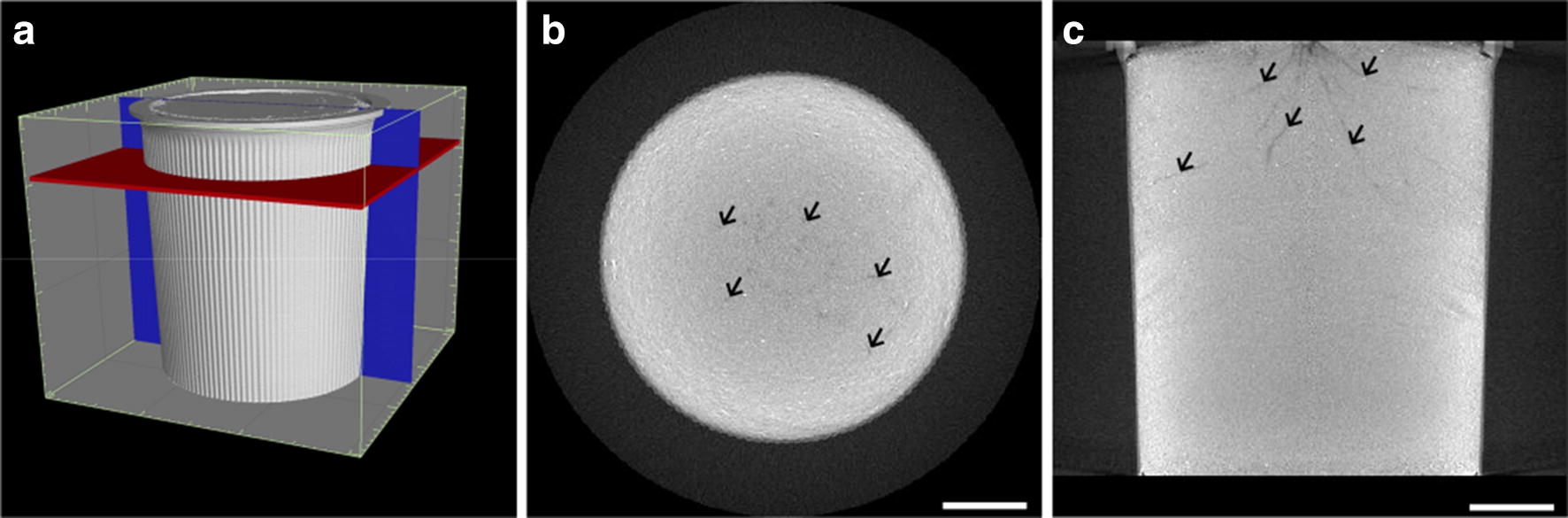
Representative X-ray CT images of a pot filled with calcined clay, using a 4-week-old Kinandang Patong as the scanning subject: **a** volume rendering with X-ray CT volume. Scale interval is 1.0 cm; **b** horizontal; and **c** vertical slices of X-ray CT volume, whose positions are indicated with red and blue rectangles, respectively, in **a**. Representative root fragments in **b** and **c** are indicated by arrowheads. Scale bar length: 5 cm

**Fig. 2:**
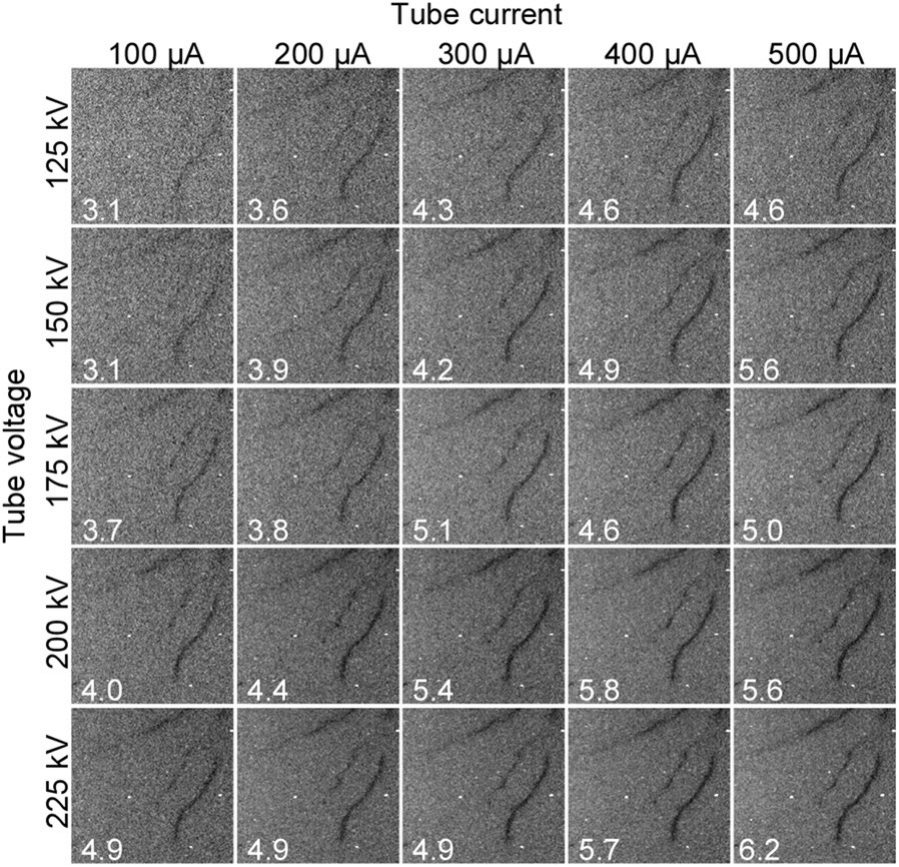
Influence of tube voltage and current on X-ray CT images, using a 4-week-old Kinandang Patong as the scanning subject. Scaled-up vertical slices are shown, using combinations of 125, 150, 175, 200, and 225 kV tube voltages, and 100, 200, 300, 400, and 500 μA tube currents. Numbers on the images indicate the contrast-to-noise ratio

As the tube voltage and current used in X-ray CT scanning affect image quality, we worked to identify the combination that exhibited the highest root-to-soil contrast. KP was grown in a growth chamber in pots filled with calcined clay, for 5 weeks, and was subjected to CT scanning—with representative CT images shown in Fig. 1. The inside of the pot was invisible in the 3-D reconstructed volumes (Fig. 1a). In the horizontal and the vertical slices (Fig. 1b, c, respectively), the roots are visible as dark pixels. The pixels of lower values were colored in black, which indicated that there was lower X-ray absorbance in the rice roots compared to that of calcined clay. To evaluate the influence of tube voltage and current on CT image quality, we scanned pots using tube voltages of 125, 150, 175, 200, and 225 kV, and tube currents of 100, 200, 300, 400, and 500 μA. The scaled-up CT slices created using these combinations are shown in Fig. 2, where it can be seen that higher voltage and current produced the highest contrast images. This was supported by the fact that the contrast-to-noise ratio (CNR) increased with higher tube voltage and current, attaining its highest value at 225 kV and 500 μA, respectively. These results conclusively indicated that a 225 kV voltage and 500 μA current were the best combination for rice root scanning, under our conditions.

### Image processing

To visualize rice root segments automatically, we developed an image processing algorithm. The process flow involved the following three steps: (1) a 3-D median filter process to increase the root-to-soil contrast; (2) an edge detection process to isolate root segments; and (3) slice stacking to construct 3-D volumes.

**Fig. 3:**
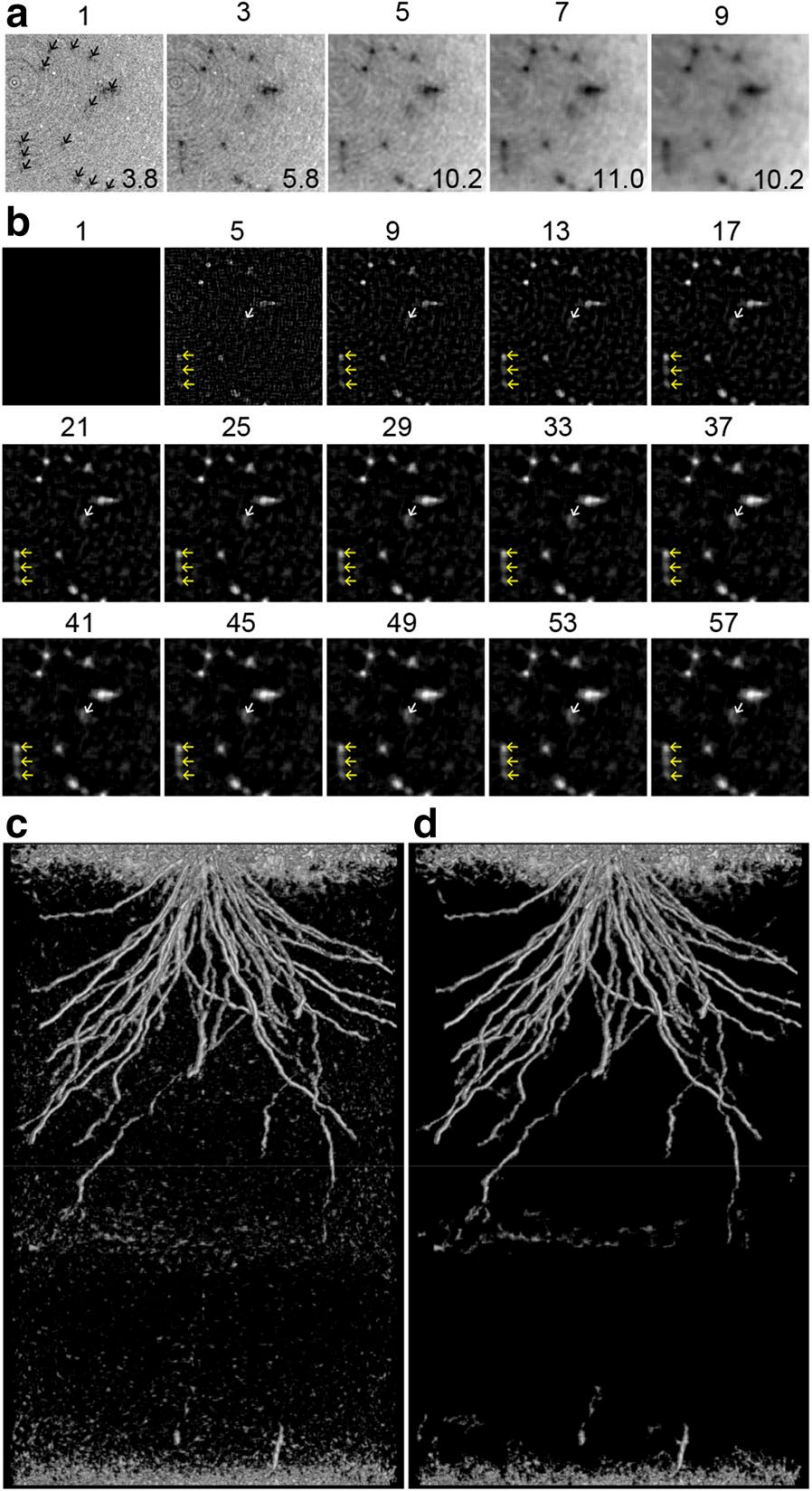
X-ray CT volume image processing, using a 4-week-old Kinandang Patong as the scanning subject: **a** scaled-up horizontal slices of an X-ray CT volume, filtered using 3-D median filters of kernel sizes 1, 3, 5, 7, and 9, with the numbers above the images indicating kernel size, and numbers on the images indicating the contrast-to-noise ratio. Arrowheads in the leftmost image indicate representative root fragments; **b** effect of blur filter kernel size on edge detection. Numbers above the images indicate the kernel size, representative root fragments are indicated by arrowheads, roots indicated by the white arrowhead are hardly visible using kernel size 5, and the roots indicated by the yellow arrowheads appear stuck together when using a large kernel size, such as 57; **c** horizontal projection of the image-processed CT volume without thresholding or size opening; **d** horizontal projection of the image-processed CT volume with thresholding and size opening

The first step was to increase the root-to-soil contrast by reducing noise in the CT image. Noise is caused by mineral particles, voids in the soil, and short scanning time, so to reduce the noise level, we applied a 3-D median filter to the CT volume. Figure 3a shows horizontal slices with five different kernel sizes—one, three, five, seven, and nine—where an image processed with the kernel size of one is equivalent to a non-filtered image. We calculated the CNR for each condition, and found it to be the highest for the 3-D median filter of kernel size seven—while the image created using the kernel size of nine was the most blurred. According to this result, we decided to use a 3-D median filter with a kernel size of seven.

The second step was to isolate root segments. CT slices that were processed using the 3-D median filter were inverted, as the CT image had soil voxels with higher value intensity and root voxels with lower value intensity. Root segments were isolated using an edge detection process that subtracted the blurred slices from their corresponding non-blurred counterparts, down to zero soil value intensity. Edge detection results achieved using various kernel sizes, from 1 to 57, are shown in Fig. 3b. Because an image blurred using kernel size one is the same as a non-filtered image, edge detection with kernel size one resulted in all-zero images. In the images processed using a kernel size > five, (Fig. 3b), signals were observed at the positions where the root could be seen in Fig. 3a. The root segments indicated by white arrowheads in Fig. 3b were not visible when kernel size five was used but visible when kernel size over 9 was used, and their size increased as the kernel size increased. Some roots, such as the root segments indicated by yellow arrowheads in Fig. 3b, were showed combined, when large kernel sizes, such as 57, were used, and the boundaries became ambiguous. Based on our observations, we decided to use kernel size 21 for edge detection in this study, as we found that when it was used, all the root signals indicated in Fig. 3a were both visible and unambiguous.

The third step was to construct a 3-D volume. All slices processed using a 3-D median filter and edge detection were stacked, while, to eliminate the influence of pot walls on RSA development, the inside regions of the CT slices were cropped. A horizontal projection and 3-D animation of the 3-D rendered volume are shown in Fig. 3c and Additional file 3, respectively. The RSA in the soil was successfully visualized, although non-root segments could also be seen in the image and the movie. Because root segmentation depends on contrast difference, all the voids in the soil were visualized. Non-root segments at the bottom were caused by the soil collapsing, while segments at the top were cracks, caused by plant growth and incompletely packed soil close to the ground surface. Small particles appearing everywhere were voids or water gradients in the soil. These small, non-root segments could be removed using thresholding and size opening processes (Fig. 3d and Additional file 4), but we did not remove them in this study as there was a risk of inadvertently erasing the small root segments of young seedlings at the same time.

We implemented the algorithm using python script (Additional file 5), and measured processing time using different hardware (Table 1). All the hardware we tested took less than 8 min for image processing, with the elapsed time dependent on the speed of the central processing unit. The fastest processing time, 2 min, was achieved using Intel^®^ Xeon^®^ E5-2650 v4. Because python is an interpreter language, batch operations were easily achieved.

**Table 1:** Image-processing times achieved using different central processing units

Central processing unit	Process count	Memory size (GB)	Processing time (min)
Intel^®^ Core™ i5-6500 CPU @ 3.20 GHz	4	16.0	8
Intel^®^ Xeon^®^ E3-1270 v5 @ 3.60 GHz	8	32.0	6
Intel^®^ Xeon^®^ E5-2650 v4 @ 2.20 GHz	48	192.0	2

The processing time included the elapsed time taken from loading the raw files to saving the processed images. Image processing was performed using python 3.7. All the CPUs were used with the multiprocessing module

### Influence of X-ray dose on rice growth

X-rays affect plant growth [25], which is a problem for 4-D RSA phenotyping using X-ray CT systems, as repeated CT scanning increases the cumulative X-ray dose. Given that tube voltage and current were constant, we identified two ways to reduce X-ray doses.

**Fig. 4:**
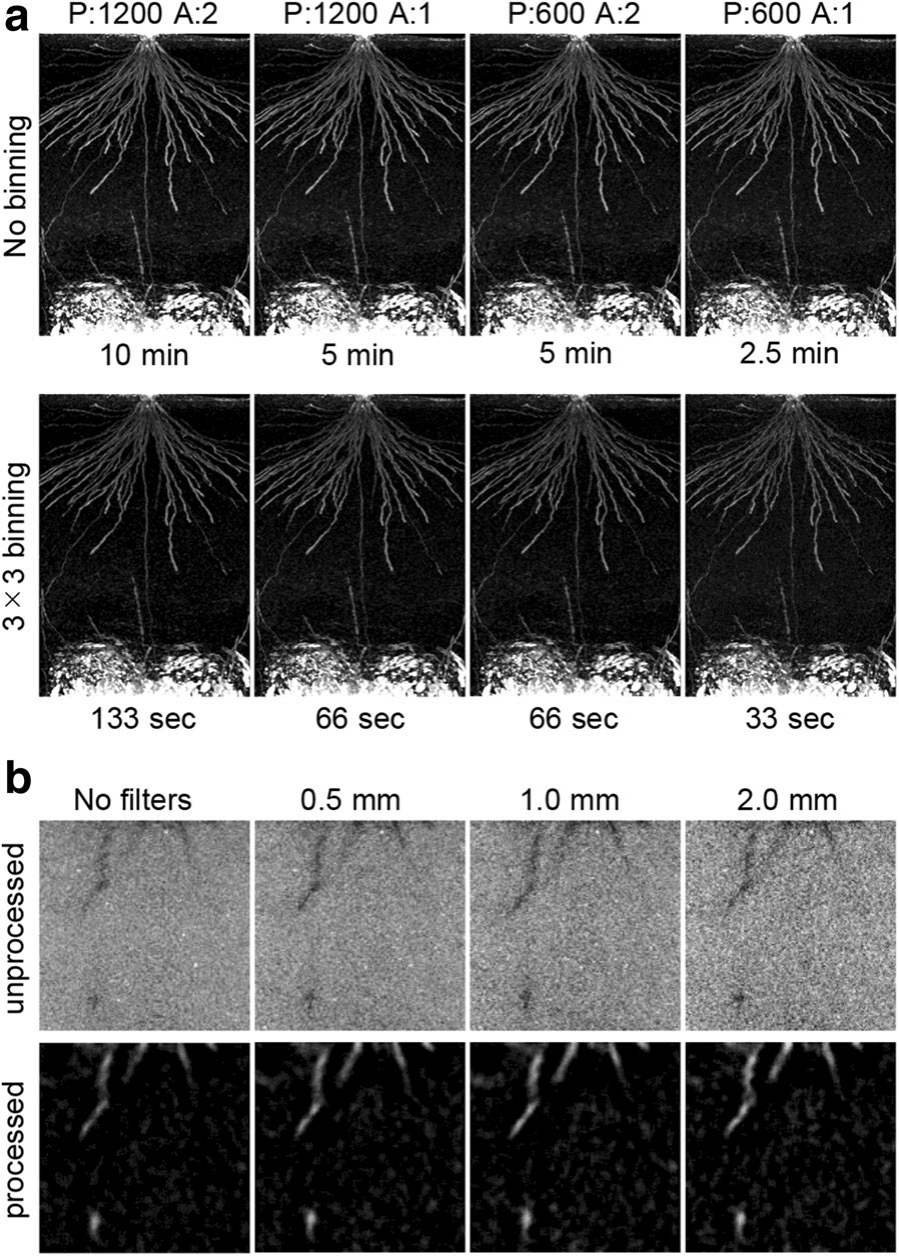
Scanning conditions to reduce X-ray dose: **a** various conditions for rapid CT scanning. An 8-week-old Kinandang Patong was scanned, using X-ray CT scanning with different scanning parameters. Horizontal projections were computed by the image-processing algorithm, and scanning times are shown below the images. P = number of projections, A = number used for signal averaging; **b** influence of Cu filters on the X-ray CT images. A pot with a 4-week-old Kinandang Patong was scanned using X-ray CT, without and with 0.5, 1.0, and 2.0 mm Cu filters. Scaled-up vertical slices of unprocessed and processed CT volumes are shown

The first way was to shorten the scanning time, applying the assumption that X-ray dose is proportional to scanning time. Figure 4a shows horizontal projections of the image-processed CT volume of a KP at 8 weeks after sowing, scanned under eight conditions and with its scanning time varied from 33 s to 10 min. We observed similar RSA in all conditions, despite the degraded image quality at faster scanning conditions. These results indicated that faster scanning was acceptable for the thick roots encountered at later growth stages, but would not be appropriate to use for the thinner roots associated with early growth stages.

The second way was by using metal filters to reduce the proportion of low-energy X-rays. Scaled-up horizontal slices of unprocessed and processed CT volumes, with and without 0.5, 1.0, and 2.0 mm copper (Cu) filters, under 10 min scanning conditions, are shown in Fig. 4b. The noise level of the unprocessed CT slices increased with increased filter thickness, while the quality of the processed CT slices obtained when using Cu filters were very similar to those achieved when no filters were used. Generally, thinner metal filters have been widely used in evaluating X-ray dose effects on plant growth—for example, 0.2 mm Cu filters have been used in rice [25], and 0.5 mm Cu filters have been used in wheat (*Triticum aestivum*), faba bean (*Vicia faba*), and barley (*Hordeum vulgare*) experiments [34, 35]. Although X-ray dose is determined by several factors, including tube voltage, tube current, and source–rotation axis distance, we considered that a 1.0 mm Cu filter would be sufficient to reduce the X-ray dose under the conditions applied in our testing.

We estimated X-ray doses using Rad Pro Dose Calculator (http://www.radprocalculator.com/). When a 225 kV tube voltage and a 500 μA current were used, the X-ray dose of the material placed 900 mm from the X-ray source, with a 0.5 mm Cu filter in place, the X-ray dose on the material was estimated as 0.55 Gy/h. With a scanning time of 10 min, and using a 1.0 mm Cu filter, the dose to rice plants was estimated to be < 0.09 Gy per scan. Because 0.09 Gy was sufficiently < 33 Gy, which has been reported as the threshold affecting plant growth [25, 36], we considered that sequential X-ray scanning did not pose a problem for rice growth. It has been reported that scanning rice daily, for 9 days with a dose of 1.4 Gy—that is, administering a total dose of 12.6 Gy—did not negatively impact rice growth [25]. If 12.6 Gy was taken as the upper limit for X-ray CT exposure, a simple arithmetic calculation indicated that 140 scanning procedures were permissible under our scanning conditions, before the rice plants would be potentially harmed.

**Fig. 5:**
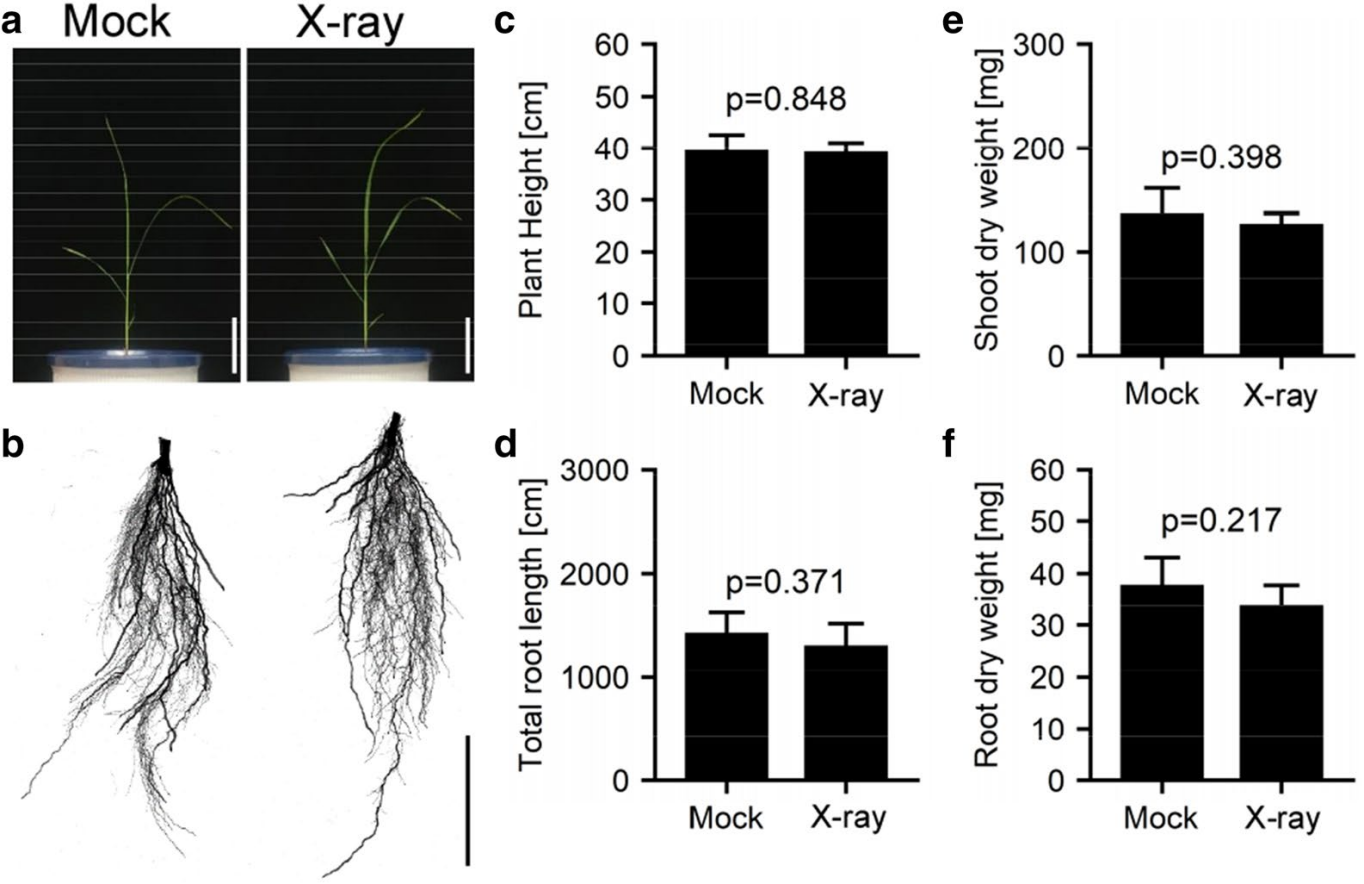
Influence of X-ray dose on plant growth. Kinandang Patong was cultivated for 2 weeks, and was subjected to daily CT scanning: **a** images of tissues above the ground (scale = 10 cm); **b** images of tissues under the ground (scale = 5 cm); **c** plant height; **d** total root length; **e** shoot dry weight; **f** root dry weight. Data for **c**–**f** were measured at 3 weeks after sowing. Sample size was five for each treatment, and error bars represent standard deviations. *P* values based on Student’s *t* tests are indicated on the figures

To evaluate the influence of sequential X-ray doses on plant growth, KP was cultivated for 2 weeks, and subjected to daily CT scanning for 7 days. The results indicated no apparent differences between shoot and root shape of mock- and X-ray-treated plants, at 21 days after sowing (DAS, Fig. 5a, b). We collected the shoot and root samples, and measured plant height, total root length of crown and lateral roots, shoot dry weight, or root dry weight (Fig. 5c–f). As a result, there were no significant differences between mock and X-ray treated plants. These results indicated that, under our scanning conditions, X-ray doses did not pose any potential impact on rice plant growth, and so, based on these results, we determined that, in our 4-D RSA phenotyping process flow, a 10 min scanning condition was appropriate for observing RSA from its early to late development stages.

### 4-D visualization of RSA development

To evaluate the developed process flow, we cultivated KP for 1 week, and then subjected it to daily CT scanning for 3 weeks. Horizontal projections of the image-processed CT volumes, and a 3-D movie, are provided in Additional files 6 and 7, respectively. From seven to 13 DAS, the root shape in the image was hazy, although daily root growth could be observed. From 14 to 20 DAS, the root shape became bolder, root length increased rapidly, and, by DAS 20, many root tips had developed beyond the imaging scope. From 21 to 27 DAS, the root shapes became increasingly bolder, while the general RSA shape remained unchanged. Overall, this test indicated that our process flow could be used for monitoring RSA development.

### Verification of root fragments in the processed CT volumes

We applied a wired basket method to verify the lengths of various diameter roots detected using our process flow. Wire baskets keep the RSA in situ when the basket is unearthed and the soil is removed, ensuring that we could collect root samples that corresponded to the root fragments observed in the X-ray CT volumes. We cultivated rice plants for 21 days, and then unearthed the baskets. Because we had observed that 21-DAS KP had many roots of different thicknesses (Additional files 6 and 7), 21-DAS rice was suitable for investigating the detection limit for root diameters. To exclude the influence of root distribution in the soil, we used three genotypes that had different RSAs [37]—KP (thick and deep-root type), IR64 (thin and shallow-root type), and Dro1-NIL (thin and intermediate-root type). Image-processed CT volume vertical projections, images shot from directly above the basket, and the vertical projection in which roots were classified by their depth and observability as different color and line types, can be seen in Additional file 8.

**Fig. 6:**
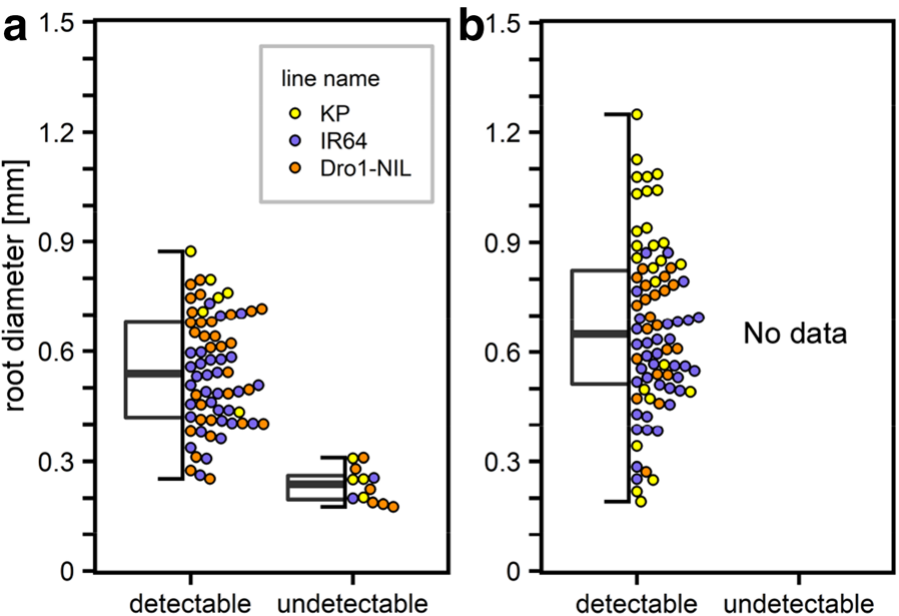
Verification of root detection limits when using the wired basket method. Kinandang Patong, IR64, and Dro1-NIL were cultivated in the wired baskets for 3 weeks. Box plots of detectable and undetectable root diameters in the processed X-ray CT volumes, for pots of diameter **a** 20 cm, and **b** 16 cm. The top and bottom of the boxes mark the first and the third quartiles, respectively. Center lines represents medians, and the whiskers show observed value ranges. The constituents of the box plot were marked as beeswarm points

We found that, when compared with the camera images, there were 68 detected and 12 non-detected roots in the image-processed CT volume. We then collected root segments and compared the root diameters of the detected and non-detected roots and found that many roots with a diameter of < 0.3 mm were not visualized, irrespective of the RSA type (Fig. 6a). As the root diameter threshold used when distinguishing the radicle and the crown roots from lateral roots was known to be approximately 0.2 mm [38], our results showed that we could not detect 15% of the roots of the 21-DAS rice plants. To visualize all radicle and crown roots, we used a smaller diameter pot (16 cm) and adjusted the source–detector and source–rotation axis distances to 800 mm and 407 mm, respectively. We repeated the wired basket assay test (Additional file 9) and found 82 detectable roots and zero non-detectable roots (Fig. 6b). The diameter of the detected roots was > 0.2 mm. In this case, we could detect almost all radicle and crown roots, with the X-ray dose per scan estimated to be 0.44 Gy. This meant that, as 12.6 Gy had been established as the safe upper limit for X-ray CT exposure, a simple arithmetic calculation indicated that plants would be unaffected by up to 28 scanning procedures. These results showed that the root detection limit could be influenced by adjusting pot diameter, source–detector distance, and source–rotation axis distance.

## Discussion

A high-throughput, non-destructive, and less-laborious RSA visualization method has been needed for isolating genes and QTLs affecting RSA plasticity. The X-ray CT system has been identified as most appropriate for this purpose, however this method has its disadvantages when applied to RSA visualization, including long scanning time, limited scanning area, long reconstruction time, and a laborious root isolation procedure. In this study, we proposed a process flow for high-throughput, 3-D RSA visualization for rice, using an X-ray CT system.

This process flow visualizes the RSA in an 18 cm diameter and 25 cm deep soil cylinder, needing just 10 min for CT scanning and 2 min for image processing. The X-ray dose was estimated at < 0.09 Gy per scan, which was suitably small, given that 12.6 Gy has been shown not to impact rice growth [25]. The CT scanning conditions in our study were as follows: each scan digitally obtained 1200 projections, using a signal averaging of two frames over 360° without binning (pixel detector resolution: 3000 × 3000), at 4.0 fps. Finally, 860 horizontal slices of pixel resolution 1024 × 1024 were computed. To harden the X-ray beam, a 1.0 mm Cu filter was used. Recent studies on rice roots that involved X-ray CT have been listed in Table 2 [39–45]. Review of these studies indicated that most studies used pots with diameter < 100 mm and required scanning times of > 1 h. This indicated that our developed process flow is suitable for 4-D RSA phenotyping, when applied to rice showing relatively large RSAs.

**Table 2:** Literature reports on use of X-ray CT for rice root visualization over the last decade

Detectable root		Pot size		Other parameters							Soil type	References
Target	Diameter [mm]	Diameter [mm]	Height [mm]	Voltage [kV]	Current [kV]	Resolution [μm]	Distance [mm]^b^	Scan time [min]	Filter	Dose [Gy]		
Main root	> 0.3	200 (180)^a^	250	225	500	300	900	10	1.0 mm Cu	< 0.09	Calcined clay^e^	This study
Main root	> 0.2	160 (140)^a^	250	225	500	200	407	10	1.0 mm Cu	< 0.44	Calcined clay^e^	This study
Main root	NA	100 (80)^a^	210 (56)^a^	120	185	40	NA	282	1.0 mm Cu	NA	Paddy soil, sieved	[39]
Main root	> 0.2	48	80	110	110	30	NA	NA	0.1 mm Cu	NA	Paddy soil, sieved	[40]
Main root	NA	80	180	180	180	60	NA	150	0.1 mm Cu	NA	Sandy clay loam soil, sieved	[41]
Main root	NA	55	100	130	240	27.3	227	73	0.1 mm Cu	1.5	Sandy loam soil, sieved	[42]
Main root	NA	72	150	180	180	50	2045^c^	7.2	NA	NA	Clay loam, sieved	[43]
Main root	> 0.4	66	108	NA	NA	200	521	75^d^	NA	NA	Play sand, sieved, or a mixture of peat-based substrate^f^ and calcined clay^g^	[44]
Main root	> 0.2	55	150	110–130	320	57.3	225	73	0.2 mm Cu	1.41	Loamy sand soil and clay loam soil, sieved	[45]

^a^Scanning area was reduced to the diameter indicated in the parentheses by cropping the images

^b^Source–rotation axis distance

^c^There was a contradiction here, in that the source–rotation axis distance was longer than the source–detector distance

^d^The value was calculated by multiplying the exposure time and the projection

^e^Profile^®^ Greens Grade™, PROFILE Products, USA

^f^Fafard #52 soil mix, Conrad Fafard Inc, USA

^g^Turface^®^, PROFILE Products, USA

Our image-processing method is fully automated and requires no operators; thus, consistent results will be obtained, no matter who processed it, and there will be significant time savings compared to situations requiring manual processing. One potential drawback is that the fully automated method may not detect obscure roots, as it performs automatic processing, with no opportunity for manual intervention. This represents a trade-off between high-throughput and accuracy, but it would be not a major problem when the objective was to observe overall RSA development.

We named this image-processing method RSAvis3D, as it facilitates the 3-D visualization of RSA development. Visualization of RSA in undisturbed field soils is a future challenge for RSAvis3D. As RSAvis3D visualizes roots as edges, all segments that create density differences, such as voids, cracks, or organic material in the soil, are visualized, and so, to reduce these false positives, we used a soil substrate with a relatively uniform particle size in our study. Another study employing feature detection of the tubular shape of roots [30] also faced this problem, and they used sieved field soil to create a uniform substrate. The system needs to be developed to the point that it can identify those segments that are roots in undisturbed field soils.

To apply RSAvis3D to other crops, soil and root type conditions need to be considered. As RSAvis3D employs intensity changes to enhance root segments, root-to-soil contrast is an important factor for efficient root isolation. Rice roots produce constitutive aerenchyma under aerobic conditions, but most field crops do not [46, 47], suggesting that rice tends to show higher contrast between its roots and the surrounding soil than other crops that do not produce constitutive aerenchyma. We need to consider the soil substrate that is available when RSAvis3D is applied to other crops.

Pot diameter also needs to be taken into account, as we found root diameter detection limits were influenced by pot diameter, source–detector distance, and source–rotation axis distance. It is known that root diameters vary widely both within and between species [48], and that root diameter generally increases as plants grow. Our work has shown that these variables must be taken into account, depending on which stage of the RSA is to be visualized.

## Conclusions

In this study, we developed a process flow for the rapid 3-D visualization of rice RSA in soil, using X-ray CT. Relatively large diameter pots, rapid scanning, low X-ray doses, and fully automatic image processing enabled the high-throughput phenotyping of RSA dynamics that is necessary in studying RSA plasticity. We have referred to the root segmentation image processing used in this process flow as RSAvis3D, and as it facilitates simple and fast processing, enhancing root value intensity, RSAvis3D has potential for use in not only visualizing, but also quantifying RSA.

## Methods

### Plant materials

Three rice (*Oryza sativa*) lines, namely, IR64 (IRGC #66970), Kinandang Patong (KP, IRGC #23364), and Dro1-NIL [37], were used in this study. KP has deep, thick roots, while IR64 has shallow, thin roots. Further, Dro1-NIL is a near-isogenic line of IR64 with a KP-type *DRO1* allele, having intermediate, thin roots.

### Growth conditions

We used Profile^®^ Greens Grade™ (PROFILE Products, Buffalo, Illinois, USA) as a soil-like root growth substrate. Profile is calcined clay and has characteristics similar to those of Turface^®^ (PROFILE Products, Buffalo, Illinois, USA) [49]. Turface is a popular substrate for root imaging using X-ray CT [44, 50]. Profile has the following advantages in studying plant roots: (1) it is hard and its volume is not affected by water content, maintaining a consistent RSA in the soil under both dry and well-watered conditions; (2) it is easily removed from the root surface, and root samples are simple to collect; (3) it can retain sufficient water and nutrients for plant growth.

Before use, Profile was rinsed with tap water three to five times and dried, as it has a quite variable labile (readily desorbable or readily plant-available) nutrient content [49]. The dried Profile was packed in the target pot, and saturated with a modified Kimura B hydroponic solution [51] consisting of 1.23 mM of NO_3_^−^, 0.41 mM of NH_4_^+^, 0.18 mM of H_2_PO_4_^−^, 1.00 mM of SO_4_^2−^, 1.78 mM of K^+^, 0.55 mM of Mg^2+^, 0.37 mM of Ca^2+^, and 8.9 μM of Fe^3+^. The pH was adjusted to 5.5, using HCl and KOH, as required. The ratio of nitrate to ammonium was based on the ratio of these compounds in the soil collected from an upland field in the Institute of Crop Science (National Agriculture and Food Research Organization, Ibaraki, Japan; 36°02′89″ N and 140°09′97″ E) on 2018 May 24, which has typically been used in the characterization of rice plant roots [52]. Except where indicated, we used custom-made, 20 cm diameter, 25 cm high pots (TSP2530P, Tecs, Itako, Ibaraki, Japan). Rice seeds were immersed in water for one d, at 15 °C and with a fungicide, and then in water for 2 days, at 30 °C. The germinated seeds were sown in the pot. Deionized water was supplied to the bottom of the pots during cultivation.

Rice plants were grown in a custom-made growth chamber (Nippon Medical & Chemical Instruments Co., Tennoji-ku, Osaka, Japan), whose temperature, light intensity, and humidity were strictly controlled. The light condition applied was to simulate 14 h of daylight, and the light intensity was set as a photosynthetic photon flux density (PPFD) of approximately 500 μmol m^−2^ s^−1^ at the top of the pot, with the exception that a PPFD of 250 μmol m^−2^ s^−1^ was applied to simulate dawn and dusk. The diurnal light intensity program was as follows: ZT0, PPFD of 250 μmol m^−2^ s^−1^; ZT1, PPFD of 500 μmol m^−2^ s^−1^; ZT13, PPFD of 250 μmol m^−2^ s^−1^; and ZT14, PPFD of 0 μmol m^−2^ s^−1^. The diurnal temperature program was based on the average diurnal temperatures for Tsukuba in July 2017, which were obtained from the Weather Data Acquisition System of the Institute for Agro-Environmental Sciences, NARO. The diurnal program was as follows: ZT0, 25 °C; ZT2, 26 °C; ZT3, 27 °C; ZT4, 28 °C; ZT5, 29 °C; ZT6, 30 °C; ZT12, 29 °C; ZT13, 28 °C; ZT14, 27 °C; ZT15, 26 °C; and ZT16, 25 °C. Humidity was set to 50% in light conditions, and 60% in dark conditions. To maintain the CO_2_ concentration, some of the air in the chamber was periodically exchanged with outside air, due to the large size of the chamber (86.4 m^3^), and CO_2_ concentration during cultivation ranged between 400 and 500 ppm.

### Soil chemical analysis

The ammonium and nitrate concentrations of the field soil were quantified by the indophenol and colorimetric methods respectively, based on diazotization with nitrate reduction. Four samples from different locations in the field were subjected to ammonium and nitrate quantification using a commercial service (Katakura & Co-op Agri Corporation, Chiyoda-ku, Tokyo, Japan).

### X-ray CT imaging and 3-D reconstruction

Rice roots were imaged in the soil using the X-ray CT system inspeXio SMX-225CT FPD HR (Shimadzu Corporation, Nakagyo-ku, Kyoto, Japan). Except where indicated, each scan digitally obtained 1200 projections, using a signal averaging two frames over 360° without binning (pixel detector resolution: 3000 × 3000), at 4.0 fps. 860 horizontal, 1024 × 1024 pixel resolution slices were computed. The final spatial resolution was 300 μm, corresponding to a total volume of 30.72 × 30.72 × 25.8 cm^3^. Tube voltages of 150, 175, 200, and 225 kV, and tube currents of 100, 200, 300, 400, and 500 μA were used. Source–detector and source–rotation axis distances were 1200 mm and 900 mm, respectively. Three Cu filters, 0.5, 1.0, and 2.0 cm thick, were used to harden the X-ray beam. Beam hardening was approximately corrected by the operating software with a correction table calculated using the correct metal material.

### Image processing

In our study, CT images were processed using Python script; the slices were loaded as 16-bit grayscale data and stacked as 3-D NumPy arrays [53]. Grayscale histograms for the CT stacks showed bimodal distributions, with one mode coming from the air fraction and the other from the soil fraction—and we roughly normalized these two modes as zero and 1024, respectively, with negative values rounded to zero. Outcomes from this were derived as follows: (1) The grayscale range for each scan was normalized, as the window width and level changed with each scan, particularly under high-power scanning conditions; (2) the normalization result was affected by the type of soil substrate, and if only one soil substrate was used, the grayscale ranges of the normalized CT volumes would all be the same; (3) the local soil fraction grayscales for single CT volumes should be different, in our study, as we used the modes for normalization. This was solved using the edge detection process described later, and allowed the soil fraction grayscales to be converted to ~ zero.

The normalized CT volume was filtered with a 3-D median, and inverted. For each slice, its blurred slice was subtracted, to isolate roots as edges. Negative values were rounded to zero, and to remove small objects, thresholding and 3-D size opening were applied to the computed 3-D volume. The processed 3-D volumes were saved as 8-bit, grayscale, 2-D images. The median, blur, and size opening filters were imported from scipy [54], opencv [55], and skimage [56] modules, respectively. Parallel processing was performed using a multiprocessing package [57], and the 25 cm high/18 cm diameter, cylinder-shaped area was trimmed to remove roots touching the pot wall. The source code for this algorithm is available in Additional file 5.

### Evaluating root-to-soil contrast

Root-to-soil contrast was evaluated using the CNR, which was defined as shown in Eq. (1):

1 $${\text{CNR}} = \frac{{\left| {S_{1} - S_{2} } \right|}}{\sigma }$$

where $S_{1}$ represents the average root fraction, $S_{2}$ stands for the average soil fraction, and $\upsigma$ represents the soil fraction standard deviation. Generally, a higher CNR means a higher root-to-soil contrast.

### Visualization of CT slices and 3-D volume

CT slices were visualized using either VG Studio MAX 3.2 software (Volume Graphics, Heidelberg, Germany), or python scripts with NumPy and matplotlib [58] modules. The 2-D image rendered from 3-D volume data was obtained using VG Studio MAX. Maximum intensity projection was obtained using python scripts, and the 3-D movies were constructed with VG Studio MAX.

### Scanning time to reduce X-ray doses

Scanning time was determined by the projection number, signal averaging number, exposing time, and binning size. In a scanning time of 10 min, each scan digitally obtained 1200 projections, using a signal averaging two frames over 360°, without binning (pixel detector resolution: 3000 × 3000), at 4.0 fps. At the fastest scanning speed, each scan digitally obtained 600 projections, using no signal averaging over 360°, with 3 × 3 binning (pixel detector resolution: 1000 × 1000), at 18.0 fps. Under these conditions, scanning was completed in 33 s. Eight scanning parameter combinations were considered, consisting of either 600 or 1200 projections, using no signal averaging or a signal averaging of two frames over 360°, with or without binning.

### Metal filters to reduce X-ray doses

Metal filters were used to reduce the X-ray doses applied to plant materials. Because longer wavelength X-rays have lower energy, their penetration ability is lower, and are thus absorbed at the material surface, resulting in higher X-ray dosage being needed there. To reduce the X-ray doses to plants, 0.5, 1.0, and 2.0 mm Cu filters were tested.

### Growth test against X-ray exposure

KP was cultivated for 2 weeks and subjected to daily CT scanning for 7 days. For X-ray treatment, KP was exposed to a 10 min X-ray scan, receiving approximately 0.09 Gy per scan. For mock treatment, KP was loaded onto the CT machine turntable, where it was left for 10 min without X-ray exposure. The positions of all plants in the growth chamber were shifted daily, to reduce the influence of position on growth. At 21 DAS, shoot and root traits were quantified: plant height was measured using a ruler, and shoot samples were collected. The shoot samples were dried, at 80 °C for 3 days, to facilitate dry weight measurement. Root samples were collected from the soil. Crown roots were separated by cutting, and all roots were spread out on the scanning tray. They were scanned using a 400-dpi scanner (Expression 12000XL, Seiko Epson Corporation, Suwa, Nagano, Japan). Total root length was measured using WinRHIZO™ Pro 2017a software (Regent Instruments, Quebec, Canada). The root samples were dried at 80 °C for three d, to facilitate dry weight measurement.

### Wired mesh basket method

The basket method was used in evaluating rice cultivar rooting angles, by counting the proportion of roots penetrating the bottom of the basket [33, 59]. As a modified approach, we used baskets (diameter: 15 cm, height: 6 cm), whose insides were woven into a mesh using *φ* 0.148 mm nylon monofilament, to keep plant RSA in situ when they were unearthed from the ground and the soil was removed. Two wire layers were included, at 1 cm and 3 cm under the top of the basket, with each wire layer consisting of seven parallel and seven perpendicular wires, spaced at intervals of ~ 1.5 cm, vertically and horizontally. The wired mesh basket was buried in the pot, and germinated rice seeds were sown on the top of the basket.

After cultivating for 3 weeks, the roots in the soil were scanned using an X-ray CT scanner, and the wired basket, including its plant and associated soil, was removed from the pot. All excavations were performed in water to avoid damaging the roots, and the soil was then removed through the basket mesh. The plant was shot from directly above, using a digital camera (Xperia Z5 Compact, Sony Corporation, Shinagawa-ku, Tokyo, Japan). To enhance root colors, the chroma of the blue and cyan colors—which were the colors of the basket, were adjusted using GIMP software (version 2.8.22, https://www.gimp.org/). By comparing the CT image and the picture of the wired basket method, we categorized all crown roots and radicles as being either detectable or undetectable by X-ray CT. The radicle and the crown roots were cut at approximately 2 cm and 5 cm from the shoot–root junction, and the resulting 3 cm root fragments were sampled. Each fragment was scanned with a 600 dpi scanner (Expression 12000XL). The average root diameter for each fragment was then calculated, using ImageJ plug-in, SmartRoot (version 4.21, [60]).

### Statistical analysis

Student’s *t* test was performed with the “t.test()” function in the R software (version 3.5.1), and a P value < 0.05 was considered significant.

## Supplementary information

**Additional file 1.** Procedure to examine soil substrates for X-ray CT scanning. Five types of soil substrates—calcined clay, volcanic ash soil, andosol, alluvial soil, and sand—were used.

**Additional file 2.** Representative CT images of rice roots in five types of soil substrates. To remove CT image noise, a minimum intensity was calculated for a 120 mm-deep CT slice, using the “thick slab” option in the VG Studio MAX software. The scale bar represented 2 cm.

**Additional file 3.** Three-dimensional animation of rice roots. A pot planted with Kinandang Patong was subjected to CT scanning. CT imagery was automatically processed using the method proposed in this paper, and the processed volumes were then animated using VG studio MAX software.

**Additional file4.** Three-dimensional animation of rice roots, using thresholding and a size opening filter. The resulting volumes were animated using VG studio MAX software.

**Additional file 5.** An example of implementing the image processing algorithm used in this study. This script was written in python3 and run in a computer using Windows 10.

**Additional file 6.** Visualization of RSA development over 3 weeks. Kinandang Patong was cultivated for 1 week and subjected to daily X-ray CT scanning. Horizontal projections of the processed CT volume are shown, with numbers above the images indicating the number of days after sowing.

**Additional file 7.** Three-dimensional animation of rice root development, from seven to 27 days after sowing. Kinandang Patong was grown for 1 week and subjected to daily CT scanning. The image-processed CT volumes were animated using VG studio MAX software.

**Additional file 8.** Representative images of the wired basket method, using 20 cm-diameter pots. CT image: vertical projection of processed X-ray CT volume; basket: image taken from directly above the basket; trace: CT image with trace lines of roots observed from the result of using the wired basket method. Lateral roots were not traced. Solid and dashed lines indicate detectable and undetectable roots in the processed X-ray CT volume, respectively. Green, blue, and red lines indicate the roots present in the soil layer between 0–1 cm, 1–3 cm, and 3–5 cm from the soil surface, respectively.

**Additional file 9.** Representative images of the wired basket method, using 16 cm diameter pots. CT image: vertical projection of processed X-ray CT volume; basket: image taken from directly above the basket; trace: CT image with trace lines of roots observed from the result of using the wired basket method. Lateral roots were not traced. Solid and dashed lines indicate detectable and undetectable roots in the processed X-ray CT volume, respectively. Green, blue, and red lines indicate the roots present in the soil layer between 0–1 cm, 1–3 cm, and 3–5 cm from the soil surface, respectively.

## Data Availability

The datasets used and/or analyzed during the current study are either included within the article (and its additional files) or are available from the corresponding author upon receipt of a reasonable request.

## Abbreviations

RSA: Root system architecture
CT: Computed tomography
QTL: Quantitative trait locus
*DRO1*: *DEEPER ROOTING 1*
KP: Kinandang Patong
3-D: Three-dimensional
4-D: Four-dimensional
fps: Frames per second
DAS: Days after sowing
PPFD: Photosynthetic photon flux density
ZT: Zeitgeber time
