# Supplementary material for: High-throughput three-dimensional visualization of root system architecture of rice using X-ray computed tomography
Source: Plant Methods. 2020 May 11;16:66. doi: 10.1186/s13007-020-00612-6 (PMC7216661; doi:10.1186/s13007-020-00612-6)
Supplement: Supplementary file 1 — Additional file 1. Procedure to examine soil substrates for X-ray CT scanning. Five types of soil substrates—calcined clay, volcanic ash soil, andosol, alluvial soil, and sand—were used. [file 13007_2020_612_MOESM1_ESM.docx]

Methods for soil selection

An upland rice cultivar, namely, Kinandang Patong was used in this study. Kinandang Patong was sown in a pot of diameter 16 cm filled with five different soil substrates, namely, calcined clay (Profile® Greens Grade^TM^, PROFILE Products, Buffalo, Illinois, USA), volcanic ash soil (collection site was unknown; sieved), andosol (Ikubyo-Shibaueyoudo, Shidara Corporation, Kanuma, Tochigi, Japan), alluvial soil (collected from a paddy field, Ibaraki, Japan; 36°02'39'' N and 140°10'65'' E; not sieved), and river sand (Kawasuna, Keiyo Corporation, Chiba, Japan). The soil volume was 3 L, and that of river sand was 2 L. The soils were saturated with Kimura B hydroponic solution [1] (0.91 mM NO_3_^-^, 0.73 mM NH_4_^+^, 0.18 mM H_2_PO_4_^-^, 1.00 mM SO_4_^2-^, 0.55 mM K^+^, 0.55 mM Mg^2+^, 0.37 mM Ca^2+^, and 8.9 μM Fe^3+^; pH 5.5) before rice sowing. Kinandang Patong was grown in a greenhouse under natural daylight conditions, i.e., air temperature and humidity were controlled (average air temperature: 30 ℃/26 ℃ at day/night; average relative humidity: 50%). Further, tap water was supplied at the bottom of the pots during cultivation.

After 23 days from sowing, each pot was scanned by an X-ray CT system (inspeXio SMX-225CT FPD HR, Shimadzu Corporation, Nakagyo-ku, Kyoto, Japan). Each scan digitally obtained 1200 projections using a signal averaging of two frames over 360° without binning (pixel detector resolution: 3000 × 3000) at 4.0 fps. Finally, 788 horizontal slices of pixel resolution 1024 × 1024 were computed. The final spatial resolution was 210 μm, which corresponds to a total volume of 21.5 × 21.5 × 16.55 cm^3^. A tube voltage of 200 kV and a tube current of 200 μA were used. The source-detector distance and the source-rotation axis distance were 800 mm and 427 mm, respectively. Further, no metal filters were used to harden the X-ray beam. Furthermore, beam hardening was approximately corrected using an operation software with a correction table calculated with metal material.

The CT images were visualized using the VG Studio MAX 3.1 software (Volume Graphics, Heidelberg, Germany). To roughly flush the noises of CT images, a minimum intensity of CT slice of depth 120 mm was calculated using the “thick slab option” in VG Studio MAX.

References

1. Yoshida S, Douglas AF, James HC, Gomez KA. Laboratory Manual for Physiological Studies of Rice. 3rd ed. Manila: International Rice Research Institute; 1976.
