# Supplementary figures and images for: High-throughput three-dimensional visualization of root system architecture of rice using X-ray computed tomography
Source: Plant Methods. 2020 May 11;16:66. doi: 10.1186/s13007-020-00612-6 (PMC7216661; doi:10.1186/s13007-020-00612-6)

## Slide 1
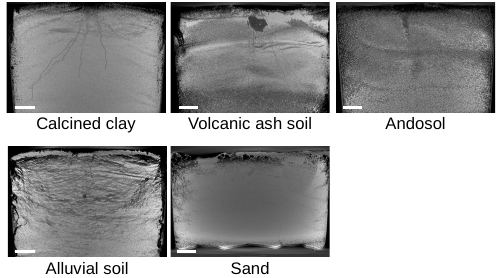

Calcined clay
Volcanic ash soil
Andosol
Alluvial soil
Sand

Supplement: Supplementary file 2 — Additional file 2. Representative CT images of rice roots in five types of soil substrates. To remove CT image noise, a minimum intensity was calculated for a 120 mm-deep CT slice, using the “thick slab” option in the VG Studio MAX software. The scale bar represented 2 cm. [file 13007_2020_612_MOESM2_ESM.pptx]

## Slide 1
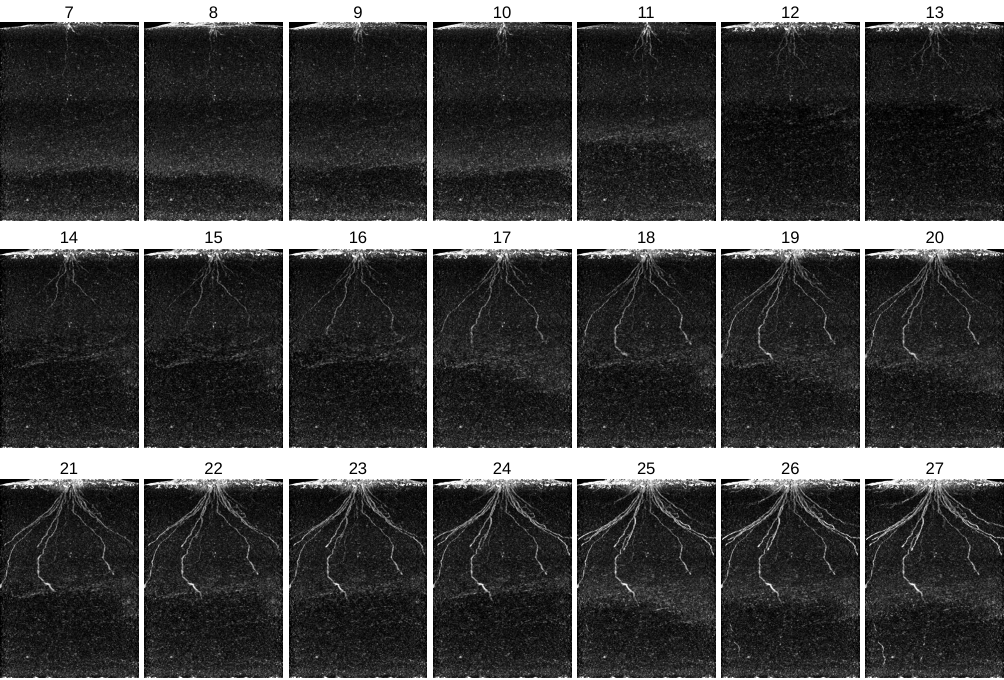

7
8
9
10
11
12
13
14
15
16
17
18
19
20
21
22
23
24
25
26
27

Supplement: Supplementary file 6 — Additional file 6. Visualization of RSA development over 3 weeks. Kinandang Patong was cultivated for 1 week and subjected to daily X-ray CT scanning. Horizontal projections of the processed CT volume are shown, with numbers above the images indicating the number of days after sowing. [file 13007_2020_612_MOESM6_ESM.pptx]

## Slide 1
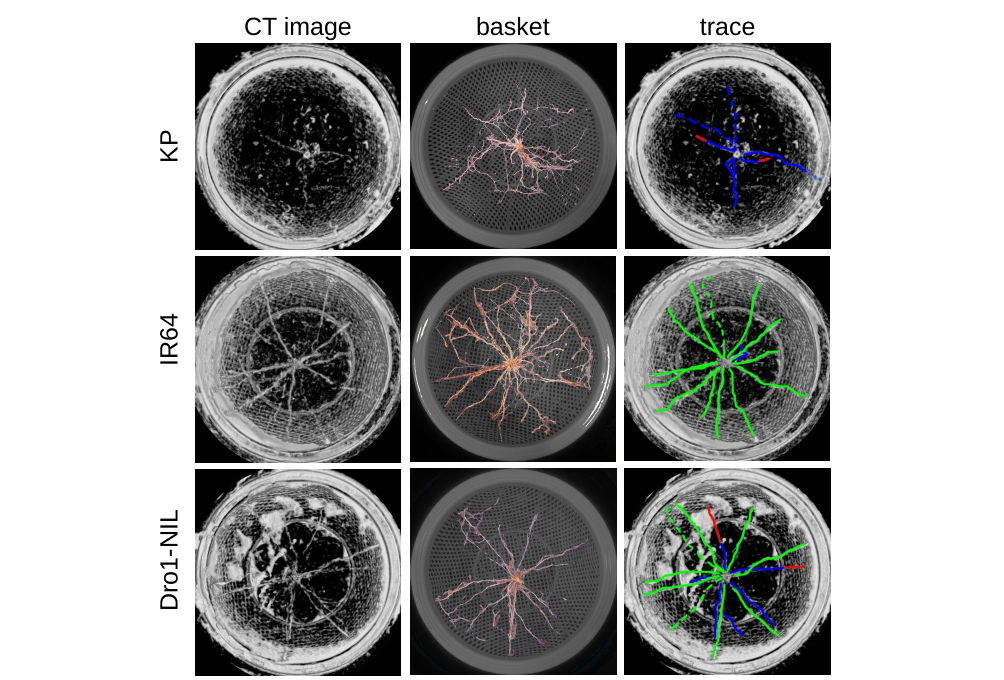

CT image
basket
trace
KP
IR64
Dro1-NIL

Supplement: Supplementary file 8 — Additional file 8. Representative images of the wired basket method, using 20 cm-diameter pots. CT image: vertical projection of processed X-ray CT volume; basket: image taken from directly above the basket; trace: CT image with trace lines of roots observed from the result of using the wired basket method. Lateral roots were not traced. Solid and dashed lines indicate detectable and undetectable roots in the processed X-ray CT volume, respectively. Green, blue, and red lines indicate the roots present in the soil layer between 0–1 cm, 1–3 cm, and 3–5 cm from the soil surface, respectively. [file 13007_2020_612_MOESM8_ESM.pptx]

## Slide 1
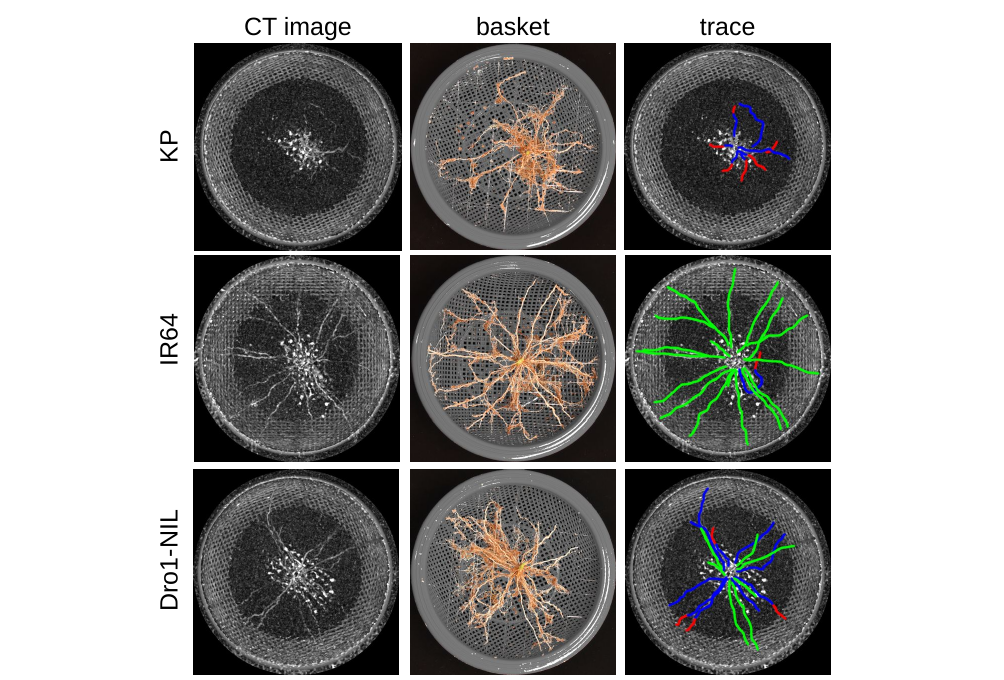

CT image
basket
trace
KP
IR64
Dro1-NIL

Supplement: Supplementary file 9 — Additional file 9. Representative images of the wired basket method, using 16 cm diameter pots. CT image: vertical projection of processed X-ray CT volume; basket: image taken from directly above the basket; trace: CT image with trace lines of roots observed from the result of using the wired basket method. Lateral roots were not traced. Solid and dashed lines indicate detectable and undetectable roots in the processed X-ray CT volume, respectively. Green, blue, and red lines indicate the roots present in the soil layer between 0–1 cm, 1–3 cm, and 3–5 cm from the soil surface, respectively. [file 13007_2020_612_MOESM9_ESM.pptx]
